# The Principles of the Voluntary Programme for the Control and Elimination of Bovine Viral Diarrhoea Virus (BVDV) From Infected Herds in Slovenia

**DOI:** 10.3389/fvets.2021.676473

**Published:** 2021-07-19

**Authors:** Ivan Toplak, Peter Hostnik, Danijela Černe, Janko Mrkun, Jože Starič

**Affiliations:** ^1^Institute for Microbiology and Parasitology-Virology Unit, Veterinary Faculty, University of Ljubljana, Ljubljana, Slovenia; ^2^Clinic for Reproduction and Large Animals-Clinic for Reproduction, Veterinary Faculty, University of Ljubljana, Ljubljana, Slovenia; ^3^Clinic for Reproduction and Large Animals-Section for Ruminants, Veterinary Faculty, University of Ljubljana, Ljubljana, Slovenia

**Keywords:** bovine viral diarrhoea virus, diagnostics, elimination, control programme, cattle

## Abstract

In Slovenia, the control of bovine viral diarrhoea virus (BVDV) infections started in 1994. Since 2014, a voluntary programme has been running according to the national rules that prescribe the conditions for recognising, acquiring, and maintaining a BVDV-free status for an individual herd. The principle is based on periodical laboratory testing and preventive measures that need to be strictly implemented in a herd. Between 2014 and 2020, a total of 348 herds were included in BVDV antibody testing, and 25.0% of tested herds were detected to be BVDV antibody positive. To recognise the BVDV-free status of the herd, the breeder should provide two consecutive tests with intervals of at least 6 months in all animals in the age from 7 to 13 months, with negative results for BVDV antibodies in ELISA. The BVDV-free status of the herd can be maintained by implementing preventive measures and can be renewed each year with one laboratory test in the age group of animals from 7 to 13 months for antibodies in ELISA. During the 7 years of the voluntary programme, 236 herds were included in the detection of BVDV in individual herds by real-time RT-PCR method and the elimination of positive animals from herds. In 71 (31.3%) herds, at least one BVDV-positive animal was detected, with the identification of a total of 267 persistently infected (PI) animals, representing an average of 2.9% of tested animals. The cost of testing for an average herd, recognised as BVDV-negative, and maintaining its BVDV-free status within the implemented voluntary programme, was €97.64/year, while for the average positive herd, the laboratory costs for elimination of BVDV were €189.59/year. Only limited progress towards eradication at the national level has been achieved in Slovenia since 2014.

## Introduction

Bovine viral diarrhoea-mucosal disease (BVD-MD) is an economically significant disease of cattle that reduces productivity and can increase death loss. It is caused by two groups of bovine viral diarrhoea viruses (BVDV): *Pestivirus A* (formerly Bovine viral diarrhoea virus 1) and *Pestivirus B* (formerly Bovine viral diarrhoea virus 2); both are members of the *genus Pestivirus*, belonging to the family *Flaviviridae* (1–3). BVDV is distributed throughout the world, with endemic areas where 70–100% of herds had detected antibodies, while in some European countries, such as Sweden, Norway, Finland, Denmark, Switzerland and Austria, the disease has been eradicated by different approaches (4–7). BVDV is spread by direct and indirect contact between cattle and causes heavy economic losses in infected herds. BVDV infection is present in persistently infected (PI) animals throughout their lifespans. The incidence of PI animals is estimated between 0.3 and 2.6% (8). PI animals are the main source of infection in infected herds, which never reach their productive potential and growth because of reduced fertility and increased susceptibility to other diseases (9). The disease can be eliminated by removing the source of infection (PI animals) from the population (5, 10). Blood tests are the most frequently used method to identify BVDV in live animals, but other samples such as skin biopsies (taken from the ear – ear notch), milk or even oral swab samples can also be collected for the detection of virus (11–15).

Slovenia is one of the smallest countries in the European Union (EU), situated in Central and South-eastern Europe, touching the Alps and Pannonia basin, bordering the Mediterranean. The total land area is 20,271 km^2^. At the end of 2019, 466,911 cattle were registered in 29,615 holdings. Most Slovenian cattle (98.3%) are reared on private family farms and 1.7% on agricultural enterprises (formerly state-owned). In 2019, an average Slovenian holding had 15.8 animals. Of the total population, 29.9% of the cattle are Simmental breed, 16.8% Holstein, 4.4% Brown and 0.9% the Slovenian autochthonous Cika breed. The rest of the animals (48.0 %) were either crossbred, cattle with unknown pedigree, or beef breeds (mostly Limousine, Charolais. or Angus). Among the active cattle population, cows predominate (34.0%), followed by calves (29.8%), heifers (20.8%), and bulls (15.4%) (16).

The monitoring of herds infected with BVDV in Slovenia started in 1994 with the identification of about 30% of infected herds, but the disease has likely been present in breeding farms for decades (13, 17). Since 1994, all bulls in breeding and artificial insemination centres are under supervision based on regular laboratory testing (18) and free of BVDV, which are important preventive measures to prevent the spread of BVDV via semen. From 1996 to 2003, from 260 to 312 breeding herds were monitored for the detection of BVDV antibodies, and the results showed that from 16.3 to 20.4% of tested animals were identified as being antibody positive (19, 20). Young bulls are tested for BVDV antibodies and the BVDV genome before entering the breeding centres and later once per year in insemination centres. However, before 2014, only a few reports of the successful elimination of BVDV from infected herds were published Slovenia (10, 13, 21).

Although it is difficult to estimate the cost of the disease because of the variable nature of the infection, many cost-benefit analyses have demonstrated the positive impact of BVDV elimination at the herd level and eradication at the national level (22–28). At the beginning of 2014, a new national rule was introduced that set out for the first time the conditions for recognising, acquiring, and maintaining a herd status of being free of BVDV in Slovenia (Uradni list Republike Slovenije no. 107/2013) (the Rule in the following text). This programme is a modification of other BVDV eradication programmes and was successfully established at the national level in Sweden, Norway, Finland, Denmark, and Austria (4, 7). The programme is run on a voluntary basis, and breeders can officially acquire the status of a herd free of BVDV. This new regulation helps Slovenian farmers decide how they want to regulate their herds' health status. The recognition, acquisition, and maintenance of status are based on the results of laboratory tests, as well as the farmer who is obliged to implement all measures to prevent the re-introduction of BVDV into the herd. Vaccination against BVDV has never been practised in Slovenia.

**Figure 1:**
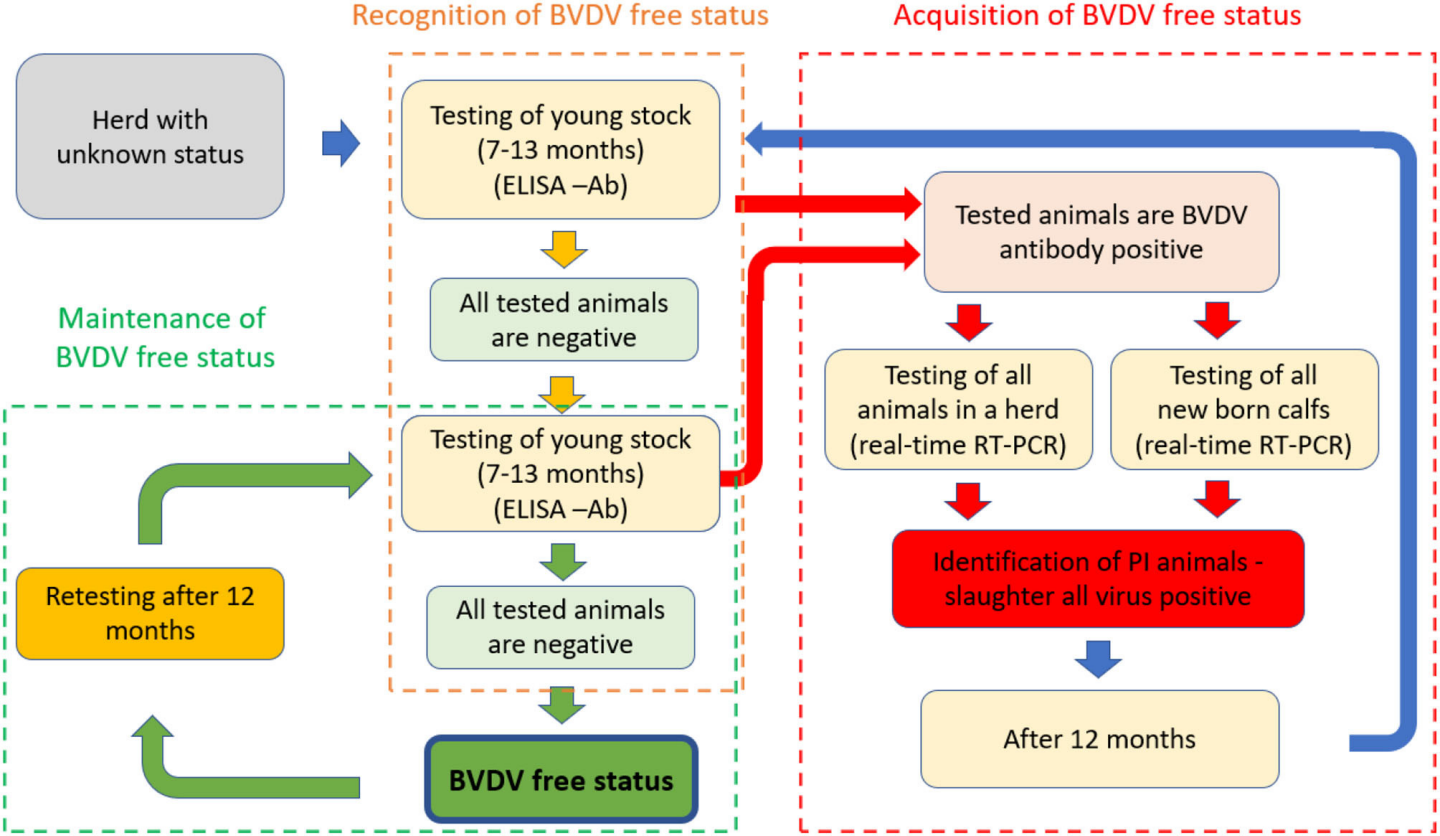
The schematic presentation of basic principles of voluntary programme for the recognition, acquisition, and maintenance of BVDV-free status in Slovenia according to the *Rule on the conditions for recognising, acquiring, and maintaining herd-free status of BVDV*.

Under the rule, the recognition of BVDV-free status in Slovenia may be achieved in 6 months if the farmer complies with the following: during the previous 12 months, no BVDV infection has been confirmed in the herd; no animal shows clinical signs of disease; animals shall be separated by a physical or natural barrier from herds with a lower status; only negative animals may be introduced into herd through quarantine; for insemination, only semen obtained from bulls free of BVDV is used. In addition to these conditions, the herd owner should provide two consecutive BVDV antibody ELISA tests at intervals of at least 6 months in all animals in the age group 7 to 13 months (Figure 1). The rule also enables the recognition of the BVDV-free status for herds without young animals; in this case, samples are collected in the next age group of animals, first in the age group from 14 to 20 months. The second sampling after 6 months and testing of young stock animals is essential in determining the stability of individual herds. If the laboratory results for all tested animals are BVDV antibody negative, the herd owner may apply for recognition of a herd free of BVDV (29).

The animals' owner applies for recognition of the status to the Regional Office of Administration for Food Safety, Veterinary and Plant Protection (AFSVPP). A list of herds free of BVDV is published on the website of the AFSVPP; it is freely available to farmers. Recognition of a herd free of BVD is granted for one year. The farmer successfully maintains the status via the implementation of preventive measures (biosecurity), and the status needs to be renewed every 12 months. To maintain BVDV-free status, the farmer should provide the laboratory results for BVDV antibodies in all animals in the herd, which are at the time of sampling aged from 7 to 13 months. The result is valid only if the tests were performed in the AFSVPP-nominated laboratory.

For BVDV-infected herds, the free status can be achieved in 18 months by the process of acquisition (set in the rule); after removing all BVDV-positive animals from the herd, the recognition of BVDV-free status is achieved through two consecutive BVDV antibody tests (Figure 1). The most important measure is the identification and elimination of all PI from the herd. In the first step, blood samples (serum) should be collected and tested from all animals in a herd to determine the presence of BVDV (identification of PI or acutely infected positive animal). The additional testing of BVDV-positive animals 14 days after the first positive results allows the differentiation of PI from an acutely infected animal; in this case, the infected animal must be kept in strict isolation until the second test result. All PI animals need to be slaughtered immediately. In addition, samples of all newborn calves (blood sample - serum) in the first week of age should be sampled in a herd during a period of one year (identification of all PI newborn calves in a herd) and tested for BVDV using the real-time RT-PCR method. All BVDV-positive calves must be culled. One year after the elimination of the last PI from the herd, all animals in the age group between 7 and 13 months are tested for the presence of BVD antibodies using ELISA. If the results of all animals are negative, the same herd is tested after 6 months again to prove the BVDV free infection in the herd. If all tested animals in the age group from 7 to 13 months are negative and farmer-implemented preventive measures (adequate biosecurity) are in place, the herd owner may apply for the official BVDV-free status (Figure 1). If it is determined that the herd has no longer qualifications for the status, the AFSVPP decides to withdraw the BVD-free status. A farm that has lost its status is deleted from the list on the website. If a farmer wants to renew the herd's status, the herd must fulfil the conditions laid down for the recognition of BVDV-free status (29).

The purpose of this study was the evaluation of the experiences obtained during the first seven years after the start of the Slovenian voluntary BVDV control programme. The principles of the programme for recognising, acquiring, and maintaining BVDV-free status were analysed according to the collected data.

## Materials and Methods

### Testing of Herds for the Detection of BVDV Antibodies by ELISA

**Figure 2:**
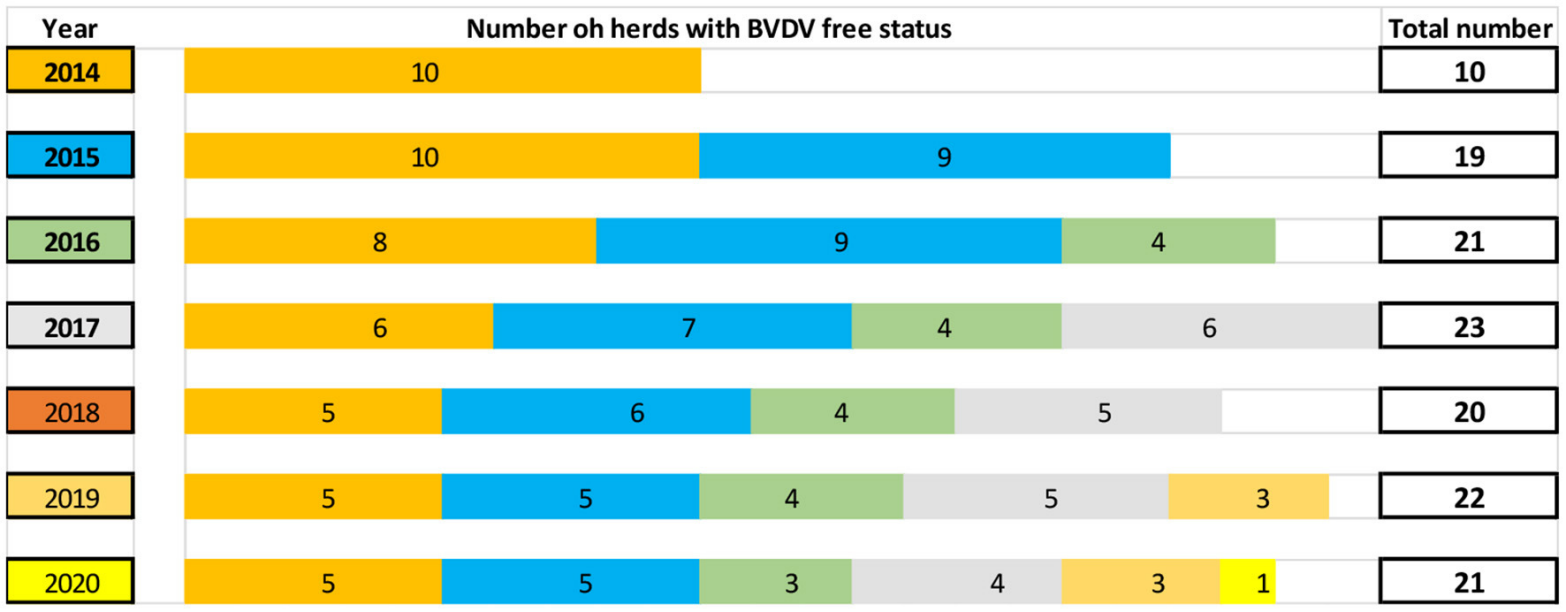
The schematic presentation of number of herds with official BVDV free status between 2014 and 2020. The same colour represents identical herds started with BVDV free status in the same year.

For recognition of BVDV-free status, a herd owner were participated voluntarily to perform two consecutive BVDV antibody ELISA testing of all animals in the age group from 7 to 13 months at intervals of at least six months (Figure 1). Between 2014 and 2020, serum samples from different herds were collected within the voluntary programme for the recognition of BVDV-free status in Slovenia. Some of those herds were sampled in consecutive years to maintain the BVDV-free status (Figure 2). The number of the collected samples from individual herds depended on the number of animals in herds in the age group from 7 to 13 months during sampling. Individual sera samples were tested using ELISA (Svanovir BVDV® Ab, Svanova, Sweden) with 100% sensitivity and 98.2% specificity of test. This ELISA was validated on Slovenian field samples, and the method has been accredited within ISO/IEC 17025 since 2007. The ELISA allows the detection of specific antibodies against BVDV for all field strains circulating in Slovenia, and the results were interpreted as positive or negative according to producer instructions. All testing for BVDV antibody detection was done in one nominated laboratory (Virology Unit, Institute of Microbiology and Parasitology, Veterinary Faculty, Ljubljana).

### Testing of Herds for Identification of BVDV-Positive Animals by Real-Time RT-PCR Method

From BVDV infected herds, samples were collected from all animals in a herd within the acquisition of BVDV-free status on a voluntary basis. Collected sera samples were tested using commercial real-time RT-PCR method for the detection of BVDV nucleic acids to identify and eliminate PI animals from infected herds. Firstly, the extraction of total RNA was performed from serum samples using a QIAamp Viral RNA kit (Qiagen, Germany), according to producer instructions, then the real-time RT-PCR was performed with virotype® BVDV RT-PCR kit (Qiagen, Indical, Germany) according to producer instruction (12). All testing for BVDV detection was done in one nominated laboratory (Virology Unit, Institute of Microbiology and Parasitology, Veterinary Faculty, Ljubljana).

Descriptive statistics were calculated using Excel (Microsoft, USA). From the laboratory price of individual serum sample testing by ELISA (€5.97 laboratory cost/sample) for the detection of antibodies and collected data over the 5 years for the tested herds, the laboratory cost of testing for an average herd was calculated. From the laboratory price of individual testing of the sample by real-time RT-PCR method (€11.84 laboratory cost/sample) for detection of nucleic acids of BVDV and collected data for tested BVDV-positive herds in the previous 5 years, the laboratory cost of testing for an average herd was calculated.

## Results

Between January 2014 and December 2020, a total of 4,756 samples were tested for BVDV antibody using ELISA and 15.8% of tested animals were detected as positive (Table 1). The lowest percentage of positive animals were detected in 2018 (11.3%) and the highest in 2019 (26.7%). The average number of tested animals for BVDV antibodies per herd was 13.63 animals between 2014 and 2020, while the average lowest number of animals (10.6) per herd was tested in 2018 and the highest (19.4) in 2014 (data not shown).

**Table 1:** Results of tested animals by ELISA for detection of antibodies within voluntary BVDV control programme in Slovenia (2014–2020).

	**Tested animals - detection of BVDV antibodies (ELISA)**				
**Year**	**No of tested animals**	**No positive**	***% positive***	**No negative**	***% negative***
2014	1,090	181	*16.6*	909	*83.4*
2015	601	75	*12.4*	526	*87.5*
2016	625	90	*14.4*	535	*85.6*
2017	557	65	*11.6*	492	*88.3*
2018	512	58	*11.3*	454	*88.6*
2019	641	164	*26.7*	477	*73.2*
2020	730	129	*17.6*	601	*82.3*
Total	4,756	762	*15.8*	3,994	*84.1*

**Figure 3:**
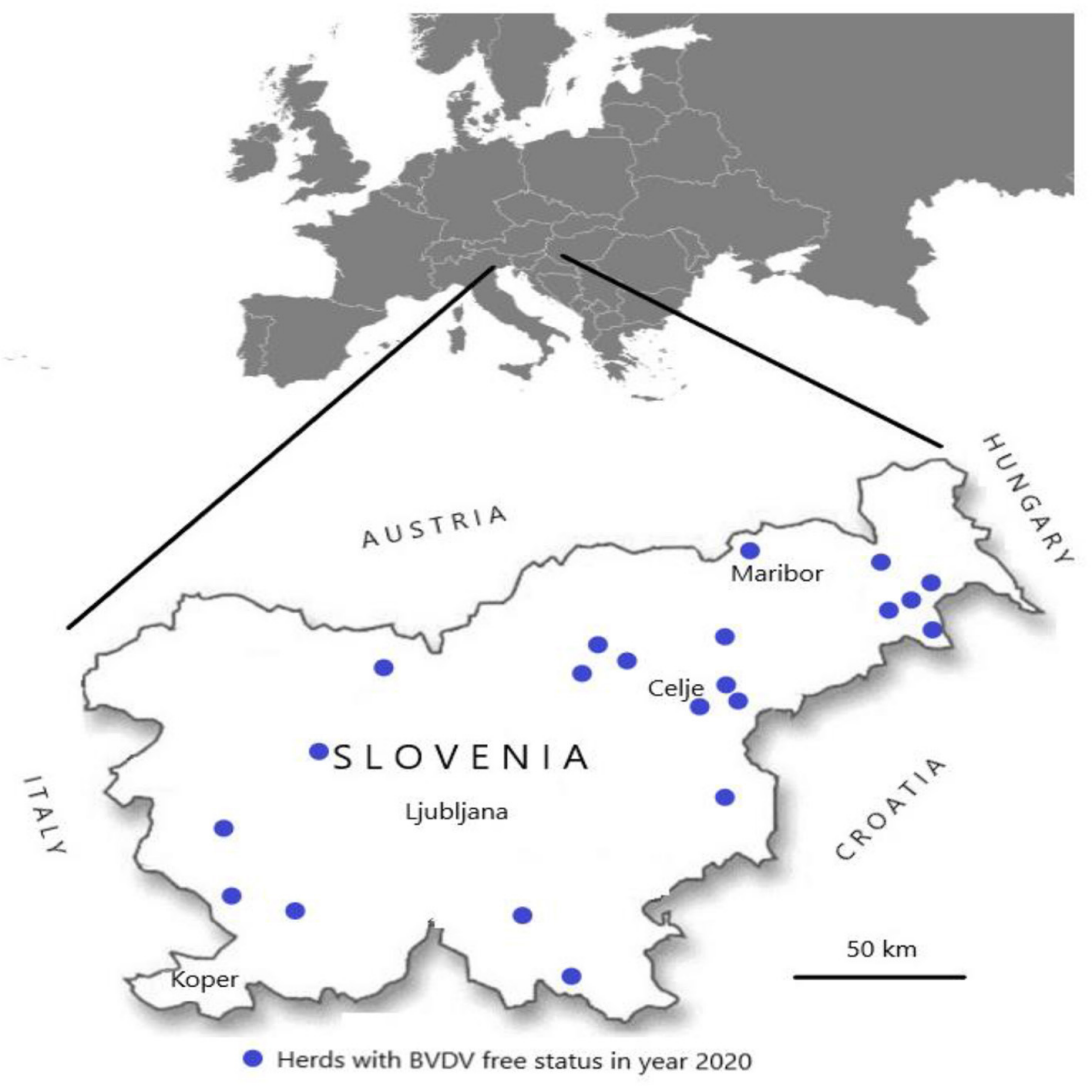
Locations of 21 herds with BVDV free status in 2020, presented on map of Slovenia.

The average laboratory cost for individual herds, calculated from data of the previous 5 years was €97.64 on a single herd/year. This calculation was consisted of two consecutive testings with ELISA at intervals of at least 6 months in all animals in the age group 7 to 13 months (€5.97 × 13.6 animals = €81.37 × 2 = €162.74) and testing of all animals in the age group 7 to 13 months once per year in next 4 years (€81.37 × 4 = €325.48). Within 5 years, the laboratory costs for an average herd with 13.6 animals are €97.64 (€162.74 + €325.48 = €488.22/5 years = €97.64 on a single herd/year). The first herd with BVDV-free status was officially confirmed on July 15th, 2014. In December 2020, official BVDV-free status was recognised for 21 individual herds, located throughout Slovenia (Figure 3).

A total of 348 herds were enrolled into testing for the recognition of BVDV-free status. and 25.0% of the herds were detected as BVDV-antibody positive (Table 2). The lowest percentage of positive herds was identified in 2019 (17.5%) and the highest in 2014 (33.9%).

**Table 2:** Results of tested herds by ELISA for the detection of antibodies within voluntary BVDV control programme in Slovenia (2014–2020).

	**Tested herds - detection of BVDV antibodies (ELISA)**				
**Year**	**No of tested herds**	**No positive**	***% positive***	**No negative**	***% negative***
2014	56	19	*33.9*	37	*66.0*
2015	51	10	*19.6*	41	*80.3*
2016	42	11	*26.1*	31	*73.8*
2017	47	12	*25.5*	35	*74.4*
2018	48	11	*22.9*	37	*78.0*
2019	57	10	*17.5*	47	*82.4*
2020	47	14	*29.7*	33	*70.2*
Total	348	87	*25.0*	261	*75.0*

Between January 2014 and December 2020, in total, 9,407 individual animals (all cattle in a tested herd and all newborn calves tested in herds) were sampled, and 2.9% (267 animals) were identified as BVDV-positive using a real-time RT-PCR method (Table 3). The lowest percentage of positive animals was detected in 2014 (2.1%) and the highest in 2018 (3.7%). The average number of tested animals for BVDV by real-time RT-PCR method per herd was 31.9 animals between 2014 and 2020, while the average lowest number of animals (20.8) were tested in 2019 and the highest (48.0) animals per herd were tested in 2014 (data not shown).

**Table 3:** Results of tested animals by real-time RT-PCR for the detection of BVDV within voluntary BVDV control programme in Slovenia (2014–2020).

	**Tested animals - detection of BVDV (real-time RT-PCR)**				
**Year**	**No of tested animals**	**No positive**	***% positive***	**No negative**	***% negative***
2014	1,882	41	*2.1*	1,841	*97.8*
2015	1,307	35	*2.6*	1,272	*97.3*
2016	1,827	46	*2.5*	1,781	*97.4*
2017	861	25	*2.9*	836	*97.1*
2018	928	35	*3.7*	893	*96.2*
2019	1,272	41	*3.2*	1,231	*96.8*
2020	1,330	44	*3.4*	1,286	*96.5*
Total	9,407	267	*2,9*	9,140	*97,0*

The average laboratory cost of testing for the identification of PI animals from BVDV-positive herd with real-time RT-PCR for individual herds calculated from data of the previous last five years was €189.59 on a single herd/year. This calculation consisted of testing of herd for BVDV antibodies (€5.97 × 13.6 animals = €81.37) to identify BVDV positive herd in the first step, then all animals in the herd were tested for PI animal identification (€11.84 × 31.9 animals = €378.40). After elimination of all PI animals from a herd, the testing at intervals of at least six months in all animals in the age group 7 to 13 months was done (€81.37 × 2 = €162.74) and the testing of all animals in the age group 7 to 13 months once per year in next 4 years (€81.37 × 4 = €325.48). Within 5 years, the laboratory costs for an average herd with 13.6 animals in the age group 7 to 13 months and 31.96 animals per average herd was €189.59 (€81.37+ €378,40 + €162.74 + €325.48 = 947.99/5 years = €189.59 on a single herd/year).

A total of 236 herds were included in the testing for the elimination of BVDV-positive animals from herds, and 31.3% of herds were detected as being BVDV-positive (Table 4). The lowest percentage of positive herds were identified in 2014 (21.7%) and the highest in 2016 and 2017 (40.0%).

**Table 4:** Results of tested herds by real-time RT-PCR for the detection of BVDV within voluntary BVDV control programme in Slovenia (2014–2020).

	**Tested herds - detection of BVDV (real-time RT-PCR)**				
**Year**	**No of tested herds**	**No positive**	***% positive***	**No negative**	***% negative***
2014	23	5	*21.7*	18	*78.2*
2015	27	10	*37.0*	17	*62.9*
2016	20	8	*40.0*	12	*60.0*
2017	30	12	*40.0*	18	*60.0*
2018	33	9	*27.2*	39	*72.7*
2019	61	15	*24.5*	46	*75.4*
2020	42	12	*28.5*	30	*71.4*
Total	236	71	*31.31*	180	*68.69*

## Discussion

The first large-scale BVDV eradication programmes were launched in Scandinavian countries in the 1990s, and the majority of Western European countries either have achieved BVDV-free status or have regional or national control programmes underway (6, 7, 30, 31). The first rule for the conditions for recognising, acquiring, and maintaining BVDV-free status of herds (the Rule) for individual herds in Slovenia started in January 2014; after seven years, only limited progress has been achieved. The basic principle of this first voluntary BVDV control based on testing of young stock for detection of BVDV antibodies proved to be correct, together with the preventive actions of farmers to prevent the re-introduction of BVDV. A total of 348 herds were included between 2014 and 2020 into BVDV antibody testing from all geographical areas of Slovenia, but only 21 herds were officially recognised as BVDV-free in Slovenia in 2020 (Figure 3). There are several reasons why more herds have not achieved BVDV free status since the beginning of the voluntary programme in 2014. According to our observation, most of the farmers included in this study started with the programme, but when they received information that the herd (laboratory testing of groups of animals between 7 and 13 months of age) was without BVDV antibodies, they halted regular laboratory testing. To them, this voluntary programme served as a tool for self-confirmation (i.e., that they are free of BVDV), and it seems that they do not see any benefit in having officially confirmed BVDV-free status.

The Scandinavian control programmes included a ban on the use of vaccine and, because vaccination against BVDV was not practised in Slovenia, the BVDV-specific antibodies are always indicative of field infection (6, 7, 31). This was very promising starting point in 2014, but a few years later, it became clearer that the voluntary programme (Figure 1) was accepted only by a limited number of Slovenian farmers. Many farms with officially recognised status of BVDV-free can successfully maintain this status from at least 3 to 7 years (Figure 2), which confirms that that the basic principle is correct and suitable for control of this disease in Slovenia's field conditions. For some other herds, which were ‘disappearing from systematic testing', it is not entirely clear why some of them just stopped with annual laboratory testing or become infected. The obtained laboratory results for herds included in this testing confirmed that the BVDV antibodies in young groups of animals are present in 15.8% of tested samples (in seven years, the average was 25.0% of identified positive cattle herds). The results of this study showed that about 75% of tested Slovenian herds within this period could have been recognised as BVDV-free (Table 2), but only 41% of them were applied for officially BVDV free status. However, according to these 21 farms with BVDV-free status in 2020, we can conclude that for most Slovenian farmers, the BVDV-free status is still not accepted as an added value of cattle health. Before starting the BVDV control programme on voluntary basis, the laboratory costs for farmers were always significant issue. This was easy adopted and resolved in only one officially nominated laboratory (located within Veterinary Faculty, University of Ljubljana), where all testing is performed, by reducing costs for laboratory tests to minimal rate (€5.97 per sample for detection of antibodies by ELISA and €11.84 per samples for detection of BVDV by real-time RT-PCR method). Through this, the calculation of costs of laboratory testing for an average herd with 13.6 tested animals/year in this study showed relatively low laboratory cost (€97.64/year/average Slovenian herd) for farmers to maintain BVDV-free status within implemented voluntary BVDV control programme. To this cost also need to be added the costs for blood sample collection, which can be variable for different size herds and need to be done by private veterinarian with concession granted by the state. Why more than three hundred cattle farmers had started with the first steps for the recognition of BVDV-free status, but later did not apply to confirm that status is not entirely clear. The dedicated protocols for official recognition of BVDV free status are freely available on the web page of AFSVPP (32) but need some administrative paperwork. With about 0.5 million cattle in Slovenia, these are owned mainly by small private family farms, with long tradition, but still not very well organised and influenced by strong competitors on the common European Union market.

Nevertheless, the owners of cattle that have already been officially recognised with BVDV-free status are confirming for the first time in Slovenia that they can successfully maintain the official status for several years; this is also valuable information for other farms that may join the programme in near future. Additional value of BVDV-free status also provides the improvement of production in the herd and reproduction parameters on a farm. The initial testing of BVDV-antibody-positive herds (animals between 7 and 13 months of age) provide immediate evidence for farmers that they have active BVDV infections in their herds and possibility to finish with elimination of BVDV through acquisition and to finish after 18 months with BVDV-free status. A similar approach is used in Scotland's national control programme with serological screening of representative young animals, known as the ‘young stock check test', indicating recent or current BVDV infection (24). Because vaccination against BVDV was never practised in Slovenia, BVDV antibody detection is result of natural infection.

The BVDV-positive herds started the process prescribed in the Rule through the identification and elimination of PI animals from a herd. During 7 years of voluntary BVDV control programme and the testing samples of cattle and newborn calves by real-time RT-PCR method, a total of 267 positive animals were identified and removed from 71 BVDV-positive herds. The BVDV-positive animals have been identified in 31.3% of tested suspected herds (the herds with positive results among young stock or herds with clinical pictures of disease or BVDV-positive results). The virus is shed by both acutely and persistently infected (PI) animals, but levels of shedding are much higher in PI cattle, which are the natural reservoir for the virus. Foetuses that become infected between 30 and 125 days of gestation and survive the infection may be born as PI calves, which are the main source of infection (33). The key point of the majority of BVDV control programmes is the early detection and the elimination of PI animals from infected herds (6, 34) BVDV-positive animals secreted virus into the environment, especially with nasal discharge, saliva, faeces, and urine (5, 8). In a BVDV-infected herd, a newborn calf can be infected within a few days or months after the birth, possibly expressing clinical symptoms due to the acute course of the disease. After 2 to 3 weeks, specific antibodies can be detected by ELISA, and those antibodies are present in the blood throughout the lifespan of the animal (5). If the mother is positive for BVDV antibodies, calves receive colostral antibodies, which remain in the blood up to 6 months, in rare cases up to 8 months of age (5). In the infected herd, BVDV is regularly transmitted between animals of all ages, and this feature of the BVDV can be successfully used as indicator of recent infection in herd by laboratory testing of age group of 7 to 13 months (22). The screening of the age group of young animals in the herd assures us with cost effective control testing, which is based on the results of detection of specific BVDV antibodies, and the actual situation regarding BVDV infection in the complete herd is recognised. During the laboratory testing, it was recognised that only a very low proportion of animals from the sampled age group from 7 to 13 months were low antibody positive in the ELISA test, usually with positive values between 10 and 20%. In these cases, the retesting of these animals after one month proved decreasing or absent of BVDV antibodies and absence of active BVDV infection in the herd, given the explanation that these first-detected antibodies were passively transferred from their mothers.

Based on the genetic comparison of identified BVDV field strains from different herds and geographic locations in Slovenia, the first molecular epidemiological study performed with BVDV isolated collected between 1997 and 2001 showed that most of the Slovenian isolated strains were of subtypes 1d and 1f (35). BVDV 2 was never detected in Slovenia. According to the phylogenetic comparison of 5'NCR and N^pro^ region of the viral genome, of 343 BVDV positive samples collected in last 20 years from 146 different herds, seven subtypes of BVDV (1a = 1, 1b = 22, 1d = 90, 1e = 8, 1f = 217, 1g = 4 and 1h = 1) were identified, providing evidence of the circulation of heterogeneous strains in Slovenia (30). The internal validations of laboratory methods within the purpose of accreditation according to ISO/IEC 17025:2017 showed that we achieved very good sensitivity and specificity for all field samples by using commercial ELISA and real-time RT-PCR methods. The sequencing of BVDV positive samples identified in positive herds within the voluntary programme and genetic comparison provides some additional data regarding the occurrence and the persistence of individual BVDV strains on our territory. The transmission of different BVDV strains between herds was observed, including the introduction of new strains into the region. This is also recognised by farmers and represent an additional risk for herds with BVDV free status. Most of the new infections in herds are result of the transmission of one of the “autochthonous” BVDV strains that have been present in Slovenia for decades, occasionally new BVDV strains are introduced into our herds, most likely with the trade of positive animals (36).

Another important preventive measure is that animals from BVDV-free herds are separated by physical or natural barriers from herds with a BVDV-unknown status, and only BVDV- and antibody-negative animals may be introduced into the herd through quarantine. Some farmers were not careful about this and lost BVDV-free status because of the introduction of one BVDV-antibody-positive animal or pregnant animal that carried a PI calf. The identified PI animals should be culled because those animals are the main source of infection for uninfected herds. The identification of PI in infected herds was successful using the protocol prescribed by the Rule; consequently, 267 new PI animals were identified in the previous 7 years. Several positive feedback responses were collected from farmers and private veterinarians a few months after the complete elimination of PI animals from infected herds, confirming that farmers are recognising the benefits of the elimination of PI from infected herds. The improvement of cattle health status was mainly detected in the calf population, where the numbers of animals with respiratory infections and diarrhoea were rapidly decreased. Although the BVDV antibody-positive animals are still present in such herds, young stock animals between 7 and 13 months of age remain BVDV-antibody-negative if the elimination of the virus was successfully completed. The identified detection of 2.9% prevalence of PI in positive herds was higher than 2.6%, as observed previously (8), but this PI prevalence in our study is related only to previously selected BVDV-positive herds; thus, the real prevalence of PI in the Slovenian cattle population is around 2%. Our voluntary BVDV programme is still missing the official tracing of culling BVDV-positive animals, and this needs to be corrected soon. Considering the estimate that that about 25% of Slovenian herds are BVDV-infected and, based on the presented data, 31.3% of infected herds have at least one PI infected animal, the total number of infected herds in Slovenia may be between 2,000 and 2,500 herds. With the identification of only 71 new positive herds, which is only a small proportion of the BVDV-infected herds (about 3%), the voluntary programme needs to be modified to a compulsory programme to achieve progress towards the eradication of BVDV infections in Slovenia.

## Conclusion

The voluntary BVDV control programme was adopted for the first time in Slovenia with the provisions for recognition, acquisition, and maintenance of BVDV-free status. The Veterinary Faculty of the University of Ljubljana offers a special package of discounts to farmers for laboratory testing of BVDV samples and have reduced the time from sample reception to the results to within one week. According to collected data of the first 7 years of running the voluntary programme, we can conclude that only a small proportion of herds have finished with BVDV-free status for the official recognition and maintenance of status. The programme is on a voluntary basis, paid exclusively by farmers; the improvement of the health status in several herds has been achieved through the implementation of the prescribed Rule. Framers who are selling and buying animals could have benefits, although the number of herds with official BVDV free status is still very low. The obtained results based on a voluntary programme have also provided some important new data regarding the prevalence of BVDV and the estimated number of positive herds. Nearby countries, such as Austria and Switzerland, have already achieved BVDV-free status at the national level. Thus, we are aware that this is only the first step starting on a voluntary basis; the next step for Slovenia will be moving to a compulsory national programme for BVDV to eradicate disease on the national level. Nevertheless, during the running of the first BVDV voluntary control programme, several cattle herds have achieved significant improvement and progress in health status following the implementation of preventive measures or have successfully maintained BVDV-free status for several years.

## Data Availability Statement

The original contributions presented in the study are included in the article/supplementary material, further inquiries can be directed to the corresponding author/s.

## Author Contributions

IT and PH: conceptualization. IT, DC, PH, JM, and JS: samples collection organisation, writing-review, and editing. IT, DC, and PH: methodology, investigation, and formal analysis. IT and PH: writing-original draft preparation. All authors have read and agreed to the published version of the manuscript.

## Conflict of Interest

The authors declare that the research was conducted in the absence of any commercial or financial relationships that could be construed as a potential conflict of interest.
